# Vitamin D Deficiency in Relapsing Idiopathic Nephrotic Syndrome in Children: Prevalence, Correlates, and Therapeutic Implications

**DOI:** 10.1155/ije/5199898

**Published:** 2025-12-31

**Authors:** Seyedeh Asma Zamani, Arash Abbasi, Behnaz Bazargani, Fahimeh Askarian, Daryoush Fahimi, Mastaneh Moghtaderi

**Affiliations:** ^1^ Hakim Children Hospital, Tehran University of Medical Sciences, Tehran, Iran, tums.ac.ir; ^2^ Children’s Medical Center, Pediatric Chronic Kidney Disease Research Center, Gene, Cell & Tissue Research Institute, Tehran University of Medical Sciences, Tehran, Iran, tums.ac.ir

**Keywords:** 25(OH)D, corticosteroids, pediatric nephrotic syndrome, proteinuria, relapse, vitamin D deficiency

## Abstract

**Background:**

Idiopathic nephrotic syndrome (NS) is a common cause of glomerulonephritis in children, often complicated by relapses and steroid dependence or resistance. Emerging evidence underscores the interplay between vitamin D metabolism and NS pathophysiology, particularly during relapse episodes.

**Objective:**

To investigate the prevalence of vitamin D deficiency in pediatric patients aged 2–12 years with relapsing idiopathic NS and to evaluate associations with proteinuria severity, relapse frequency, and steroid exposure.

**Methods:**

This cross‐sectional study included 100 children diagnosed with idiopathic NS and at least one documented relapse. We enrolled all NS patients with completed follow‐up for 1 year. Serum 25‐hydroxyvitamin D (25[OH]D) levels were measured during relapse and six months later. Clinical and biochemical data, including proteinuria, albumin, creatinine, and corticosteroid usage, were analyzed.

**Results:**

At relapse, 84% of patients were vitamin D deficient, with only 7% reaching sufficiency after 6 months. A significant inverse correlation was observed between 25(OH)D levels and both proteinuria (*r* = −0.62, *p* < 0.001) and relapse frequency (*r* = −0.48, *p* < 0.01). Corticosteroid dosage was not significantly associated with vitamin D recovery.

**Conclusion:**

Vitamin D deficiency is highly prevalent among children with relapsing idiopathic NS and correlates with greater disease activity. Routine monitoring and timely supplementation of vitamin D may be essential components in managing NS flares and preventing long‐term complications.

## 1. Introduction

Nephrotic syndrome (NS) represents a prototypical glomerular disorder. It remains one of the most frequent causes of chronic kidney disease in children, accounting for a substantial global healthcare burden due to its relapsing course, complications, and long‐term sequelae [1]. The hallmark clinical features of NS include nephrotic‐range proteinuria (> 40 mg/m^2^/hr), hypoalbuminemia, generalized edema, and hyperlipidemia [2]. Among the etiological subtypes, idiopathic NS—predominantly minimal change disease (MCD)—accounts for over 85% of cases in children, with the majority showing initial responsiveness to corticosteroids [3, 4]. However, a significant number of them (approximately 50%) experience frequent relapses (frequently relapsing NS, FRNS) or become steroid dependent (SDNS) or steroid resistant (SRNS), necessitating intensified immunosuppressive therapy and increasing the risk of chronic kidney injury [5, 6]. The pathophysiology of NS involves disruption of the glomerular filtration barrier, primarily at the level of the podocytes, resulting in selective or nonselective proteinuria. Podocyte integrity is increasingly recognized as a critical determinant of disease progression and response to therapy [7]. At the molecular level, recent insights reveal the involvement of dysregulated immune responses, T‐cell dysfunction, cytokine imbalances, and altered expression of slit diaphragm proteins such as nephrin and podocin in driving proteinuria and relapse phenomena [8, 9]. Vitamin D—traditionally known for its potential in calcium‐phosphate homeostasis—is now acknowledged as a pleiotropic hormone with profound effects on innate and adaptive immunity, podocyte biology, and systemic inflammatory modulation [10, 11]. In the context of NS, both the disease process and treatment modalities contribute synergistically to a state of functional hypovitaminosis D. Massive urinary loss of vitamin D binding protein (DBP), secondary to glomerular leakiness, leads to a significant reduction in the serum levels of 25‐hydroxyvitamin D [25(OH)D], the primary circulating biomarker for vitamin D status [12, 13]. Moreover, long‐term corticosteroid use suppresses the synthesis of osteocalcin, impairs calcium absorption, induces osteoblast apoptosis, and accelerates bone resorption—further depleting vitamin D reserves [14, 15]. Emerging evidence also highlights the role of the vitamin D receptor (VDR), a nuclear transcription factor expressed in podocytes, renal tubular cells, and immune cells, in mitigating renal injury. Activation of VDR by calcitriol or its analogs exerts antiproteinuric, antiapoptotic, and anti‐inflammatory effects through downregulation of transforming growth factor‐β1 (TGF‐β1), suppression of nuclear factor‐κB (NF‐κB), and modulation of the Th17/Treg immune axis [16–18]. Several investigations and early‐phase clinical trials have demonstrated that vitamin D analogs such as paricalcitol and alfacalcidol reduce proteinuria and glomerulosclerosis in both diabetic and nondiabetic kidney disease models [19, 20]. VDR polymorphisms, including FokI, BsmI, and ApaI, have further been implicated in determining susceptibility to NS, responsiveness to steroids, and relapse frequency in diverse pediatric populations [21, 22]. Despite the accumulating body of literature underscoring the renal‐protective and immunoregulatory roles of vitamin D, data specific to its dynamics during acute relapse episodes of idiopathic NS in children remain scarce. Most available studies focus on baseline levels or post‐treatment outcomes, with limited granularity on intrarelapse variations and their clinical significance. Therefore, the current study is designed to bridge this knowledge gap by investigating the prevalence of vitamin D deficiency in children aged 2–12 years experiencing a relapse of idiopathic NS and by exploring its correlations with clinical severity, proteinuria levels, frequency of relapse, and corticosteroid exposure. This inquiry may offer critical insights into the pathobiological relevance of vitamin D during active disease and inform evidence‐based strategies for timely intervention.

## 2. Methods

### 2.1. Study Design and Setting

This hospital‐based, cross‐sectional analytical study was conducted at the Pediatric Nephrology Unit of a tertiary referral hospital from January 2020 to December 2021. The study protocol received approval from the institutional review board and ethics committee (approval ID: IR.TUMS.IKHC.REC.1399.412).

### 2.2. Study Population

Eligible participants were children aged 2–12 years with confirmed idiopathic NS according to KDIGO criteria, presenting with at least one documented relapse during the study period. Exclusion criteria included patients with secondary causes of NS, such as lupus nephritis and Henoch–Schoenlein purpura, chronic systemic illnesses, including diabetes mellitus and autoimmune disorders, congenital anomalies of the kidney and urinary tract, or recent intake of pharmacological doses of vitamin D supplementation exceeding 1000 IU/day within the preceding 3 months to minimize confounding variables. Some patients that had not completed follow‐up were excluded from this study.

### 2.3. Operational Definitions

Relapse was defined as the reappearance of proteinuria measuring 3+ or greater on a urine dipstick for three consecutive days, proteinuria exceeding 40 mg/m^2^/hr, or a ratio of protein to creatinine in a spot urine sample was greater than 0.2. Remission was characterized as proteinuria below 4 mg/m^2^/hr or negative/trace dipstick results for 3 consecutive days, or a ratio of protein to creatinine in a spot urine sample was less than 0.2. Vitamin D status of assessed according to Endocrine Society guidelines, with deficient levels defined as below 20 ng/mL, insufficient levels between 20 and 30 ng/mL, and sufficient levels at 30 ng/mL or higher. Frequent relapse is defined as occurring two or more times in 6 months or four or more times during 1 year.

### 2.4. Sample Size and Sampling Technique

A calculated sample size of 100 participants provided greater than 80% power to detect significant correlations between serum 25(OH)D levels and relapse frequency, using a two‐tailed alpha of 0.05. Consecutive sampling was utilized to enroll all eligible patients meeting the inclusion criteria during the study period.

### 2.5. Data Collection Instruments and Variables

Patient information was gathered using a structured data abstraction form capturing demographic data, including age, sex, height, weight, and body surface area, along with clinical history encompassing age at diagnosis, number and timing of relapses, hospitalization frequency, and corticosteroid regimen details. We evaluate the vitamin D level at least 6 months after the start of NS. Laboratory investigations comprised serum 25‐hydroxyvitamin D levels measured using standardized ELISA kits, urinary protein excretion assessed through 24‐h collection and spot urine protein‐to‐creatinine ratio, and serum creatinine, albumin, total cholesterol, calcium, phosphate, and parathyroid hormone measured using automated analyzers following standard protocols. Free 25(OH)D level could be a better biomarker of vitamin D status than total 25(OH)D level, but in our laboratory, only the total 25(OH)D level was measured.

### 2.6. Statistical Analysis

Data were entered into SPSS version 22.0 and verified for accuracy and completeness. Continuous variables were presented as mean ± standard deviation or median with interquartile range as appropriate, while categorical variables were summarized using frequencies and percentages. Independent *t*‐tests or Mann–Whitney *U* tests were applied for two‐group comparisons, with ANOVA or Kruskal–Wallis tests used for multiple group analyses. Associations between 25(OH)D levels and continuous variables were assessed using Pearson’s or Spearman’s correlation coefficients. Categorical comparisons utilized chi‐square or Fisher’s exact tests. Multivariate linear regression was performed to adjust for potential confounders, with statistical significance set at a two‐sided *p* value below 0.05.

### 2.7. Quality Control and Bias Mitigation

Laboratory assays were performed in duplicate with intertechnician variability minimized through blinded assessments. All measurements occurred in the same clinical laboratory under standardized environmental conditions. Confounding was reduced through strict adherence to inclusion/exclusion criteria and adjustment during multivariate analyses.

### 2.8. Ethical Considerations

The study followed the Declaration of Helsinki principles, with patient data confidentiality maintained throughout all study phases. Participants received standard clinical care regardless of study involvement.

## 3. Results

### 3.1. Participant Characteristics

A total of 100 children with idiopathic NS (INS) were enrolled in the study, comprising 58 males (58%) and 42 females (42%), with a mean age of 8.06 ± 2.7 years (range: 2–12 years). The mean duration of the disease since initial diagnosis was 2.1 ± 1.3 years. The average number of relapses in the preceding year was 2.72 ± 1.1 episodes per patient.

### 3.2. Vitamin D Status at Baseline and Follow‐Up

At the time of relapse, serum 25‐hydroxyvitamin D (25[OH]D) levels were markedly reduced, with a mean value of 6.22 ± 3.1 ng/m. Based on Endocrine Society criteria, 84% of participants were classified as vitamin D deficient (< 20 ng/mL), while the remaining 16% had insufficient levels (20–30 ng/mL). None of the patients had sufficient vitamin D levels (≥ 30 ng/mL) at baseline.

Six months following the relapse episode and initiation of standard corticosteroid therapy with supportive care, there was a statistically significant increase in mean 25(OH)D levels to 14.13 ± 4.7 ng/mL (*p* < 0.001). Despite this improvement, only 7% of patients achieved sufficiency, 44% remained insufficient, and 49% continued to have deficient levels.

### 3.3. Proteinuria and Biochemical Trends

The mean urinary protein excretion (24‐h collection) at the time of relapse was 13.03 ± 5.2 mg/dL, which decreased to 3.68 ± 2.3 mg/dL at follow‐up (*p* < 0.001). Serum albumin showed a corresponding increase from a mean of 2.1 ± 0.6 g/dL to 3.7 ± 0.5 g/dL (*p* < 0.001). Additionally, serum total cholesterol levels, elevated at relapse (mean: 274 ± 48 mg/dL), decreased significantly over the study period (mean: 193 ± 39 mg/dL, *p* < 0.001).

### 3.4. Correlation Analyses

There was a strong negative correlation between baseline serum 25(OH)D levels and proteinuria severity (*r* = −0.62, *p* < 0.001). Similarly, a moderate inverse relationship was observed between 25(OH)D levels and annual relapse frequency (*r* = −0.48, *p* < 0.01). Serum albumin levels positively correlated with 25(OH)D concentrations (*r* = 0.51, *p* < 0.01), suggesting that hypoalbuminemia may co‐contribute to reduced bioavailability of vitamin D.

### 3.5. Subgroup Analysis

When stratified by sex, age groups (2–5, 6–8, and 9–12 years), and corticosteroid dose categories (low, moderate, and high cumulative dose over the prior 6 months), there were no statistically significant differences in vitamin D recovery trajectories (*p* > 0.05 for all comparisons). Similarly, ethnicity and urban vs. rural residence did not significantly affect vitamin D status or relapse outcomes.

### 3.6. Multivariate Analysis

In the adjusted multivariate linear regression model, baseline proteinuria (*β* = −0.49, *p* < 0.001) and serum albumin (*β* = 0.37, *p* = 0.004) remained independent predictors of serum 25(OH)D levels after controlling for age, sex, corticosteroid exposure, and BMI.

### 3.7. Adverse Events

No patients experienced hypervitaminosis D or hypercalcemia during the study period. No serious adverse events attributable to study measurements or sampling were reported.

### 3.8. Summary

The results demonstrate a persistently high burden of vitamin D deficiency in children with relapsing INS, with only partial improvement despite standard treatment. Significant associations between hypovitaminosis D, proteinuria severity, and disease relapse rates underscore the potential utility of vitamin D monitoring and supplementation in this population. Table 1 demonstrates the participant characteristics such as their gender, age, mean duration of their disease, and relapses. Table 2 shows the vitamin D level at the baseline and follow‐up. Table 3 shows the amount of proteinuria and serum level of albumin and cholesterol. In Table 4, we demonstrate the correlation and analyses between serum vitamin D and the relapses.

**Table 1:** Participant characteristics.

Characteristic	Value
Total participants	100
Male participants (%)	58%
Female participants (%)	42%
Mean age (years)	8.06 ± 2.7
Age range (years)	2–12
Mean duration of disease (years)	2.1 ± 1.3
Mean number of relapses (episodes/year)	2.72 ± 1.1

**Table 2:** Vitamin D status at baseline and follow‐up.

Timepoint	Mean serum 25(OH)D level (ng/mL)	Vitamin D deficiency (%)	Vitamin D insufficiency (%)	Vitamin D sufficiency
Baseline (at relapse)	6.22 ± 3.1	84	16	%
6‐Month follow‐up	14.13 ± 4.7	49	44	%

**Table 3:** Proteinuria and biochemical trends.

Metric	Baseline (relapse)	6‐month follow‐up	*p* value
Urinary protein excretion (mg/dL)	13.03 ± 5.2	3.68 ± 2.3	< 0.001
Serum albumin (g/dL)	2.1 ± 0.6	3.7 ± 0.5	< 0.001
Serum total cholesterol (mg/dL)	274 ± 48	193 ± 39	< 0.001

**Table 4:** Correlation analyses.

Correlation	*r* value	*p* value
Baseline 25(OH)D vs proteinuria severity	−0.62	< 0.001
Baseline 25(OH)D vs annual relapse frequency	−0.48	< 0.01
Serum albumin vs baseline 25(OH)D	−0.51	< 0.01

## 4. Discussion

This study demonstrates that vitamin D deficiency is highly prevalent among pediatric patients with INS relapses and correlates significantly with disease activity markers, including proteinuria severity and relapse frequency [23].

Multiple mechanisms may account for the profound depletion of 25‐hydroxyvitamin D (25[OH]D) observed during relapse episodes. First, the loss of DBP in urine due to increased glomerular permeability results in a direct reduction of circulating vitamin D levels [24]. DBP serves as the primary carrier of vitamin D and its metabolites, and its depletion may hinder both transport and renal reabsorption mechanisms [3]. Second, corticosteroid therapy—while essential in inducing remission—negatively impacts vitamin D metabolism by promoting bone resorption, reducing intestinal calcium absorption, and impairing hepatic hydroxylation of cholecalciferol [25]. Third, the systemic inflammatory milieu associated with active NS may upregulate fibroblast growth factor 23 (FGF23), which suppresses renal 1‐alpha‐hydroxylase and limits calcitriol synthesis [26].

The significant inverse correlation between baseline 25(OH)D levels and both proteinuria and relapse frequency supports the hypothesis that vitamin D status may be a modifiable factor in disease severity. Previous research by Guido Gembillo et al. and Molina et al. has shown that vitamin D supplementation, particularly with active analogs like calcitriol or paricalcitol, can reduce urinary protein excretion and stabilize glomerular architecture [14, 27]. Our findings support these results in a real‐world pediatric context and suggest that moderate improvements in vitamin D levels are associated with clinical benefit.

Additionally, the positive correlation between serum albumin and 25(OH)D levels observed in our study is biologically plausible. Since 25(OH)D is primarily protein‐bound (mainly to DBP and albumin), hypoalbuminemia may exacerbate the bioavailability deficit, further limiting the endocrine and autocrine functions of vitamin D [28]. This underlines the importance of considering albumin‐corrected vitamin D indices or direct measurement of free 25(OH)D in future studies.

While subgroup analyses in our study revealed no statistically significant differences in vitamin D recovery based on age, sex, corticosteroid dose, or residential background, these findings should be interpreted with caution due to potential sample size limitations and unmeasured behavioral or nutritional confounders. Moreover, the short follow‐up duration (6 months) limits the ability to assess long‐term outcomes such as growth velocity, bone mineral density, or progression to chronic kidney disease.

Notably, VDR polymorphisms such as FokI, BsmI, and ApaI have been linked to variations in NS susceptibility and treatment response in various ethnic populations [29]. In Charan V S’s study in 2024, children with a first episode of NS had significant vitamin D deficiency. The free and bioavailable vitamin D levels are measured in these children during the proteinuric phase [30] in children with NS to validate the utility of bioavailable vitamin D in clinical practice. Although we did not perform genotyping in this study, future prospective trials incorporating genetic profiling could offer insight into personalized vitamin D therapy in NS management.

Our findings provide a strong rationale for integrating routine vitamin D screening into the clinical algorithm for relapsing pediatric NS. Additionally, consideration should be given to proactive supplementation strategies—potentially involving active analogs or higher‐dose cholecalciferol regimens—to achieve sufficiency, mitigate immune dysregulation, and reduce disease recurrence.

### 4.1. Limitations of the Study

Our study is associated with our previous study about vitamin D levels in the first episode of NS. Our study has some limitations such as a small sample size and a lack of PTH and bone densitometry. Free 25(OH)D level is a better biomarker of vitamin D status than total 25(OH)D level, but in our laboratory, only the total 25(OH)D level was investigated. Nutrition and diet behavior are confounder points of our study.

In these prior studies, DBP levels were not measured. Of note, total 25(OH)D and free/bioavailable 25(OH)D levels are associated, and it is possible that a larger sample size would have enabled us to detect a weak relationship between total 25(OH)D and BMD. Prior studies that found this relationship generally had sample sizes greater than 200, and when correlation coefficients between 25(OH)D and BMD were reported, they were less than 0.24.

## 5. Conclusion

This study underscores the substantial burden and clinical relevance of vitamin D deficiency in children experiencing relapses of INS. Despite standard corticosteroid‐based remission induction and a 6‐month follow‐up period, a significant proportion of the cohort remained vitamin D‐deficient or insufficient, suggesting persistent disruptions in vitamin D metabolism beyond the acute phase of the disease. The strong inverse correlations observed between serum 25‐hydroxyvitamin D (25[OH]D) levels, proteinuria, and relapse frequency provide compelling evidence that vitamin D status may not only reflect disease severity but also influence its trajectory.

These findings highlight the need for a paradigm shift in the management of pediatric INS, whereby vitamin D assessment and correction become integral components of routine clinical care. Given its multifaceted roles in immune modulation, podocyte stabilization, and suppression of profibrotic and proinflammatory cytokines, ensuring vitamin D sufficiency may serve as an adjunctive therapeutic target to reduce disease activity and improve renal outcomes. Moreover, considering the interplay between hypoalbuminemia, DBP loss, and impaired bioavailability of vitamin D, tailored strategies—such as higher dosing, active analog administration, or measurement of free vitamin D—may be warranted in severe or refractory cases.

Incorporating vitamin D optimization into existing treatment algorithms holds the promise of synergizing with immunosuppressive therapies to better control relapses and potentially lower the risk of progression to chronic kidney disease. However, this approach must be validated through multicenter randomized trials that assess long‐term outcomes, optimal dosing regimens, and the impact of genetic and nutritional modifiers.

Vitamin D is far more than a skeletal micronutrient in the context of NS—it is an immunomorphological modulator whose deficiency represents both a marker and a mediator of disease activity. Its strategic assessment and management may redefine the standards of care in pediatric glomerular disease.

## Ethics Statement

The authors declare that all procedures contributing to this work comply with the ethical standards of the relevant national guidelines on human experimentation and have been approved by the institutional ethics committee. Our patients were under 16 years of age, and all parents or guardians were informed and signed an informed consent form before the study.

The method was approved in compliance with scientific and ethical standards. All procedures were performed in line with the relevant guidelines and regulations. The Medical Ethics Committee of Tehran University of Medical Sciences approved this study.

The corresponding author “Mastaneh Moghtaderi” affirms that this manuscript is an honest, accurate, and transparent account of the study being reported; that no important aspects of the study have been omitted; and that any discrepancies from the study as planned.

## Disclosure

All authors have read and approved the final version of the manuscript.

## Conflicts of Interest

The authors declare no conflicts of interest.

## Author Contributions

This study was a project done by Seyedeh Asma Zamani, supervised by Mastaneh Moghtaderi, Fahimeh Askarian, Daryoush Fahimi, Behnaz Bazargani, Arash Abbasi, and Mastaneh Moghtaderi provided cases to the study. Mastaneh Moghtaderi had full access to all of the data in this study and takes complete responsibility for the integrity and accuracy of the data and analysis.

## Funding

No funding was received for this study.

## Data Availability

The data supporting this study’s findings are available on request from the corresponding author. The datasets created during the current study are not publicly accessible due to the possibility of compromising individuals’ privacy.
